# Long-term exposure to more frequent disturbances increases baseline carbon in some ecosystems: Mapping and quantifying the disturbance frequency-ecosystem C relationship

**DOI:** 10.1371/journal.pone.0212526

**Published:** 2019-02-21

**Authors:** Brian Buma, Thomas Thompson

**Affiliations:** 1 Department of Integrative Biology, University of Colorado, Denver, United States of America; 2 USDA Forest Service, Resource Monitoring and Assessment Program, PNW Research Station, Anchorage, AK, United States of America; University of the Chinese Academy of Sciences, CHINA

## Abstract

Disturbance regimes have a major influence on the baseline carbon that characterizes any particular ecosystem. Often regimes result in lower average regional baseline C (compared to those same systems if the disturbance processes were lessened/removed). However, in infrequently disturbed systems the role of disturbance as a “background” process that influences broad-scale, baseline C levels is often neglected. Long-term chronosequences suggest disturbances in these systems may serve to increase regional biomass C stocks by maintaining productivity. However, that inference has not been tested spatially. Here, the large forested system of southeast Alaska, USA, is utilized to 1) estimate baseline regional C stocks, 2) test the fundamental disturbance-ecosystem C relationship, 3) estimate the cumulative impact of disturbances on baseline C. Using 1491 ground points with carbon measurements and a novel way of mapping disturbance regimes, the relationship between total biomass C, disturbance exposure, and climate was analyzed statistically. A spatial model was created to determine regional C and compare different disturbance scenarios. In this infrequently disturbed ecosystem, higher disturbance exposure is correlated with higher biomass C, supporting the hypothesis that disturbances maintain productivity at broad scales. The region is estimated to potentially contain a baseline 1.21–1.52 Pg biomass C (when unmanaged). Removal of wind and landslides from the model resulted in lower net C stocks (-2 to -19% reduction), though the effect was heterogeneous on finer scales. There removal of landslides alone had a larger effect then landslide and wind combined removal. The relationship between higher disturbance exposure and higher biomass within the broad ecosystem (which, on average, has a very low disturbance frequency) suggest that disturbances can serve maintain higher levels of productivity in infrequently disturbed but very C dense ecosystems. Carbon research in other systems, especially those where disturbances are infrequent relative to successional processes, should consider the role of disturbances in maintaining baseline ecosystem productivity.

## Introduction

Disturbance processes, like fires, windstorms, and landslides are ubiquitous and present in all ecosystems [1, 2] and critical to carbon cycling [3]. They trigger rapid change in resources availability, short term carbon loss [4], and reorganization of the ecological community [5]. Individual biomes and ecosystems generally have a characteristic suite of disturbance processes operating on varying spatial and temporal scales, and individual ecosystems can be maintained by characteristic disturbance regimes [6]. While historical systems may have had some sort of characteristic C stock value which incorporated the historical disturbance regime, e.g. [7], climate change is rapidly altering the drivers of disturbances, increasing or decreasing their frequency, intensity, and subsequent severity. Novel disturbance processes and new interactions between historical disturbances are also emerging [8]. As a result, understanding the fundamental relationship between disturbances and carbon is important to projecting the magnitude and even the direction of future changes in carbon stocks.

Knowledge of how disturbances alter ecosystem C stocks from short to long time scales has advanced considerably over past decades. Long-term data, paleoecological reconstructions of historical disturbances, e.g., [9], and real-time flux measurements [4] have allowed for precise understandings of C loss, recovery, and net change. However, most studies have been conducted in single disturbance scenarios. Our knowledge of multiple disturbances and their interactive effects on C stocks and trajectories is much more limited [8, 10].

Over relatively short time spans, and holding other factors constant, relative differences in C stocks are driven by the mean return interval of a disturbance and the time required to recover lost C from that disturbance [11, 12]. If disturbances are frequent (occur prior to the recovery of C lost in earlier events), the cumulative effect would be C stocks less than the “potential” maximum C stocks, e.g. [13]. Very long-term chronosequences, however, suggest that at millennial or longer return intervals the relationship between disturbances and baseline ecosystem C is reversed [14], where baseline means average C densities across multiple sites. Undisturbed ecosystems sometimes display retrogression, where soil nutrient availability declines [15] result in lowered ecosystem productivity and potentially biomass C [16], for example through paludification [17]. Disturbance events can also serve to increase nutrient availability by rapidly decomposing and releasing nutrients sequestered in live or dead biomass [18, 19] (but see [20]), disrupting impermeable soil layers [21, 22], exposing unweathered parent material [23], or providing openings for early successional, N-fixing species [24]. Overall, intermediate frequencies of disturbance may result in more productive forests [25] and potentially higher overall C than in the absence of disturbance events. So what is the fundamental role of disturbance frequency in ecosystem C balance?

The prediction of lowered C at both very high disturbance frequencies and very low disturbance frequencies results in the expectation of maximal “baseline” biomass for a given ecosystem occurring at relatively intermediate levels of disturbance frequencies when examined over very long periods of time [11, 16]. Time between disturbance events would be long enough (on average) that biomass stocks could re-accumulate after any given disturbance event, but not so long as to result in hydrologically driven productivity declines, nutrient limitations, or other retrogressive process.

Support for the “disturbance-baseline C hypothesis,” that an intermediate level of disturbance promotes higher baseline biomass, is typically inferred from chronosequences [14, 16] or paleo ecological studies [11, 22]. An alternative method involves investigating biomass and ecosystem functions along an exposure gradient. Similar to a chronosequences varying time via space, this method varies disturbance frequency as a function of space.

Our goal here is to test the disturbance-baseline C hypothesis, and in particular the untested expectation that areas of very infrequent disturbance will have less baseline C than areas with intermediate frequency disturbance events, indicating that the disturbance process is influential in maintaining long-term productivity and higher biomass levels. It is difficult to generate long-term, spatially explicit records of disturbance frequencies. Many disturbance types interact directly with landscape composition and structure, shaping the relative frequency and disturbance intensity themselves (e.g., fire and vegetation feedbacks).

### Site

The temperate rainforests of southeast Alaska avoid these difficulties. The disturbance regime is spatially stable with little confounding effects. Event size is small relative to landscape size and recovery rapid relative to return interval, so the assumption that recent large events are unlikely to be driving any observed pattern is reasonable [26, 27]. There is essentially no fire; the most recent evidence of widespread natural fire is >5000 years ago and limited to the southern portion of the region [28]. Insect and pest outbreaks are infrequent and minor, rarely resulting in mortality [29]. The primary disturbances are windstorms and mass movement, driven and constrained by topography. Yellow-cedar decline, a mass mortality event associated with climate warming [30, 31] is widespread but does not appear to be affecting net C stocks [32]. As a result, it is possible to test if long-term spatial differences in disturbance exposure (representing the relative frequency of disturbances within a given area, *sensu* [33]) drives spatial differences in regional ecosystem C stocks above and beyond the short-term fluctuations resulting from any given disturbance event and recovery.

Wind is the most common disturbance driver. The location of low-pressure systems and their movement is strongly constrained by the semi-permanent Aleutian low pressure system, the shape of the Gulf of Alaska, and the Coast Range mountains. This constrains storm force winds to the south-southeasterly aspects [34], and the incised landscape results in a heterogeneous distribution of exposed slopes. Dendrochronological reconstructions show that disturbance frequency corresponds well with exposure [35], a pattern exploited to study soil C dynamics [36], watershed-scale C distributions [37], and regional forest dynamics [32].

Landslides (and avalanches) are similarly spatially stable. Because the region has relatively shallow soils the most dominant type of landslides are shallow debris avalanches and flows that occur in predictable locations (associated with slope, drainage, and wind) and a several-century return interval [38]. For both processes, because the topography of the landscape has been essentially stable since the Last Ice Age, exposure to is assumed to be relatively consistent.

The climate is moderate along the entire coast due to its hyper-maritime nature. The species composition is consistent, dominated by intermixed conifer species, *Picea sitchensis* and *Tsuga heterophyla*, with other species occasionally found throughout: *T*. *mertensiana*, *Thuja plicata*, and *Cupressus nootkatensis*. *Alnus viridis* is the only major broadleaf species, and generally only found in recently disturbed locations. This consistent species assemblage limits confounding C estimates with changes in community composition. All species (except *T*. *plicata* found in the far south) range both further north and south of the region limiting concerns about significant range edge effects and shifting dominance patterns [39, 40]. Therefore the relationship between relative disturbance exposure and C stocks can be quantified spatially with minimal confounding influence from climate or variable vegetation composition.

Here we test the disturbance-baseline carbon hypothesis that higher disturbance rates are associated with higher C in infrequently disturbed systems and lower C in frequently disturbed areas. This was done by asking three primary questions:

1. Does long-term disturbance exposure drive fundamental differences in biomass, density, or basal area at the regional scale, above and beyond any influence of short-term variability driven by recent disturbance or stochastic mortality?

2. Does the type of disturbance change the magnitude of the effect?

3. What is the cumulative baseline non-soil ecosystem C in the region (excluding human management), and how does that compare to scenarios without disturbance?

## Methods

USFS Forest Inventory and Analysis plots (FIA) is the most extensive forest survey in the region. FIA plots are a fixed area design, consisting of one central location and three surrounding subplots at 120^0^, 240^0^, and 360^0^, at a 36 m center-to-center distance from the central point; all four subplots are 7.3m radius. Plots are placed approximately 5.3 km apart and visited on ~10 year intervals (10% of plots visited per year). Survey dates utilized here range from 2005 to 2017. Plots with any history of management, historical or contemporary, were discarded to focus on natural dynamics and total potential regional C in the absence of human intervention. In total, 1491 plots met this criterion. Of those, 484 are in non-forested locations (cover permanently <10%). For testing the disturbance exposure-carbon relationships, only the forested plots were utilized (n = 1007) unless otherwise noted. This eliminates non-informative low values of C in areas on glaciers and other alpine areas. The entire set of 1491 plots were used for regional C modeling to accurately capture low C values at high elevations.

Three forest structure metrics were assessed. The FIA program estimates dry biomass by measuring all live tree greater than 2.54 cm diameter at breast height (DBH) and dead trees >5” DBH; allometric equations are used to scale volume to dry biomass [41]. Where needed, this value was converted to C at a rate of 50% [42]. Trees per hectare (TPH) is a significant response variable because a decrease in per-tree biomass may be offset by an increase in density. Finally, while C content or biomass is generally the goal of ecosystem ecology studies in a global context, estimation of those values requires allometric equations which embody considerable uncertainty. Basal area (BA) is a variable which incorporates both tree diameter and tree density and may be more precise for plot to plot comparisons [43]. All three variables were assessed using identical methods.

### Environmental variables

At each site, several topographic, bio-climatic, and disturbance exposure metrics were quantified. Elevation, slope, and transformed aspect [44] were derived from 1 arc-second ASTER GDEM2 data. Mean summer and winter precipitation and temperature, mean annual temperature, and the mean length of growing season (LOG), date of continuous freeze (DOF), and date of continuous thaw (DOT) were taken from the Scenarios Network for Arctic Planning (1960–1990 climate normal, 771m, [45]), then downscaled to 30m resolution via bilinear interpolation. Percent forest cover was from the Landsat Vegetation Continuous Fields (VCF) tree layer [46], which estimates the percentage of ground per 30m pixel covered by vegetation >5m in height via rescaling MODIS vegetation values with Landsat 5 TM and ETM+ data.

### Disturbance exposure

Exposure represents the relative probability of a disturbance within a given spatial area (e.g., [47]). Wind exposure was modeled via methods described in [33] (the EXPOS model), modified [35] and applied [32] to the region. Briefly, the method assumes straight-line winds which interact with topography; high topographic barriers upwind can shelter downwind locations. Wind passes over those topographic obstacles and bends downwards at eight angles ranging from 1 – 14^o^ in 2^o^ increments. The degree of exposure is a result of how directly a location is exposed to storm winds. Using the observed distribution of wind directions [34], an average of three incoming wind directions (SW to SE) was created to determine average long-term exposure.

**Fig 1:**
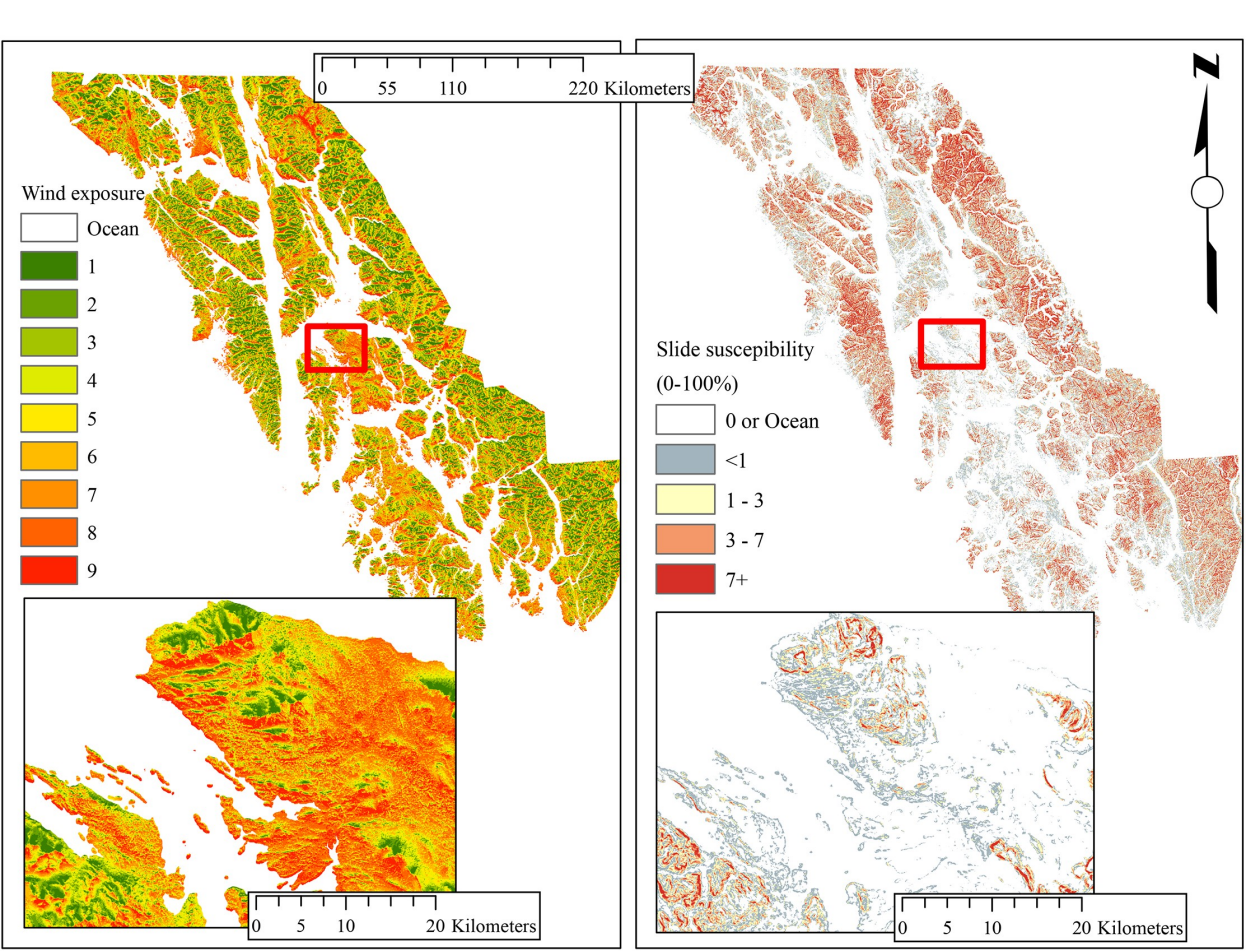
Disturbance exposure maps utilized in the investigation. Both are relative scales, from low to high. Wind exposure refers to storm force winds, see [35] for design and comparison to field data. Slide susceptibility is based on observed slides, see [48] for design. Inset maps are for illustrative purposes at a finer scale.

A recent assessment [48] modeled landslide likelihood, ranging from 0 (very unlikely) to 100 (has slid/likely to slide), based on observed slide locations in non-harvested forest throughout the central portion of the region. Significant variables include slope, local topographic position, contributing area, and exposure to wind during storms. The two exposure maps (Fig 1) were created at 30m resolution in R using the raster, sp, and rgdal packages.

### Analysis

In these forests, stochastic mortality of large trees can cause very high variance at plot scales, as single large trees (>60m tall) may dominate a plot [43, 49]. The fundamental interest here is the underlying “baseline” average, incorporating that natural variability. In other words, the effect of time-since-disturbance was intentionally not considered. Thus 1) the modeling and statistical focus is on relative differences *on average* compared to exposure and 2) all models were chosen and explicitly tuned to avoid overfitting plot-scale data (with its inherent time-since-disturbance variability) to regional projections.

Several methods were used because of the inherent variance in the data given the goals, but no test was considered as definitive on its own. This avoids the potential for Type 1 errors resulting from multiple methods to influence results. The statistical framework and all modeling choices were made *a priori*, without seeing the real data. All coding was done in R using dummy data by Buma, then the framework was applied to the actual FIA dataset by Thompson independently. This avoids unintentional bias or over-tuning in modeling building.

#### Direct correlation

To explore direct relationships between disturbance exposure and biomass at the plot scale, simple, single-error linear regressions were constructed between the disturbance exposure metrics (wind, landslides) and the response variables (biomass, TPH, BA). Assumptions were checked and data log transformed if necessary.

#### Disturbance importance, plot scale

Two stepwise regression models were conducted, one with, and one without, the disturbance variables (wind and landslide exposure). Backwards stepwise linear regression was done, starting with all the potential explanatory variables. When the best model (as evaluated via AIC reduction) was constructed for each condition (with and without considering disturbance), Chi-square difference tests were used to compared between the models with and without disturbance. Significant differences in residual deviance were interpreted as one model being significantly better.

#### Spatial modeling

Finally, random forest modeling was used to explore variable significance and create predictive maps of regional C. Random forests [50] are a machine learning technique built on classification and regression trees that accommodate non-linear interactions and correlated/non-independent predictor variables. To construct the random forests, first a random subset (80% of the data) were selected for model construction with 20% retained for testing.

The extreme topography, which stretches from the ocean to icefields, required a two-step modeling procedure to capture both variation *within* forested areas as well as variation *between* forested areas and non-forested areas. First, a random forest model for the forested areas only (>10% cover, n = 1007) was created, which focused on variation as a function of variables relevant to intraforest variability in the structural attributes. Then, to constrain biomass estimates in areas of non-forest or low forest cover (<10% tree cover), a second model incorporating the full suite of plots (n = 1451) was constructed, which results in a clear high elevation/ice cap vs. forest distinction. This output was applied to areas on the landscape where tree cover was <10% to create a continuous response surface.

Random forests have a well-known tendency to overestimate low values and underestimate high values due to a regression to the mean associated with bagging predictors. A correction was applied [51] using cubic smoothing splines.

Using four different biomass calculation methods, [52] determined that the aboveground component measured by the FIA data is between 46–58% of the total biomass C. Data was scaled by those factors for a high and low estimate (“scaling” factor in Table 1). To accommodate the steep topography, final values were scaled from raster pixel area to actual surface area by a cos(slope) raster.

**Table 1:** Estimated potential carbon content. Scaling refers to the assumed proportion of total ecosystem carbon in the modeled biomass.

Scenario	Scaling (%)	Estimated C (Pg)	Difference from actual (%)
Actual conditions	58	1.21	-
	46	1.52	-
No wind	58	1.19	-2
	46	1.50	-2
No slide	58	0.98	-19
	46	1.24	-18
Neither	58	1.02	-16
	46	1.29	-15

#### Disturbance importance, regional scale

To estimate the significance of disturbances to regional C balance, we utilized a method conceptually similar to [53], which “turned off” fire in a global vegetation distribution model and calculated differences between with-fire and without-fire outputs. To estimate the cumulative effect of wind on the landscape, the model was re-run with wind exposure set to 1 (the minimum) across the entire landscape. The difference between this “null” wind model and the biomass model was calculated via subtraction. The same was done for landslide likelihood (set to 0) and both wind and landslides simultaneously to create three hypothetical comparison scenarios–without wind, without landslides, and without both.

## Results

There were direct correlations between exposure to both disturbance exposures and the forest variables considered, though with considerable variability. Higher wind exposure was significantly correlated with higher tree density, though with considerable variance (F_(1, 1005)_ = 23.4, p < 0.001, r^2^ = 0.02), but not biomass or BA. Landslide exposure was significantly associated with all three metrics, in the direction of larger, more widely spaced individuals–higher landslide exposure was correlated with significantly higher biomass (F_(1, 1005)_ = 137.6, p < 0.001, r^2^ = 0.12) and BA (F_(1,1005)_ = 104.4, p < 0.001, r^2^ = 0.09), and significantly lower tree density (F_(1, 1005)_ = 14.4, p < 0.001, r^2^ = 0.01).

### Disturbance importance, plot scale

Models incorporating disturbance as a predictor were significantly improved over non-disturbance models. For dry biomass, model selection identified the candidate model which included landslide exposure as significantly better than the model without (Chi_2_ test, p < 0.001). Wind was not retained in the stepwise variable selection process, and thus no significant improvement was seen for dry biomass when considering wind. For BA, both wind and landslide exposures were retained in the better model (Chi_2_ test, p <0.0001). For TPH, both wind and landslide exposures were retained in the better model (Chi_2_ test, p < 0.0001).

### Spatial modeling

Modeled dry biomass was correlated with the 20% of observations retained for testing (simple linear regression, comparison between predicted and independently observed dataset; F_(1,200)_ = 133.3, r^2^ = 0.4, p < 0.001). Basal area was similar to dry biomass (F_(1, 200)_ = 97.4, r^2^ = 0.33, p < 0.001). Tree density observations were also significantly correlated with model predictions, though with high variance (F_(1, 200)_ = 7.8, r^2^ = 0.04, p < 0.006).

After applying the spline correction and projecting over the region, the fit between predicted and observed values at each sampling point was strong for dry biomass (F_(1, 1005)_ = 1509, p < 0.001, r^2^ = 0.60), tree density (F_(1,1005)_ = 1311, TPH, p < 0.001, r^2^ = 0.57), and BA (F_(1,1005)_ = 1863, p < 0.001, r^2^ = 0.65). Incorporating the entire dataset, including non-forested plots, further improved the overall predicted vs. observed fit for all variables (F_(1, 1489)_ for all; dry biomass: r^2^ = 0.69; TPH: r^2^ = 0.67; BA: r^2^ = 0.77). The most important variables in predicting variation in biomass and BA within forested landscapes was observed forest cover, slope, elevation, and likelihood of landslide initiation. For tree density, the most important variables were observed forest cover, aspect, elevation, and mean winter precipitation.

#### Estimated regional carbon, basal area, and tree density and disturbance effects

**Fig 2:**
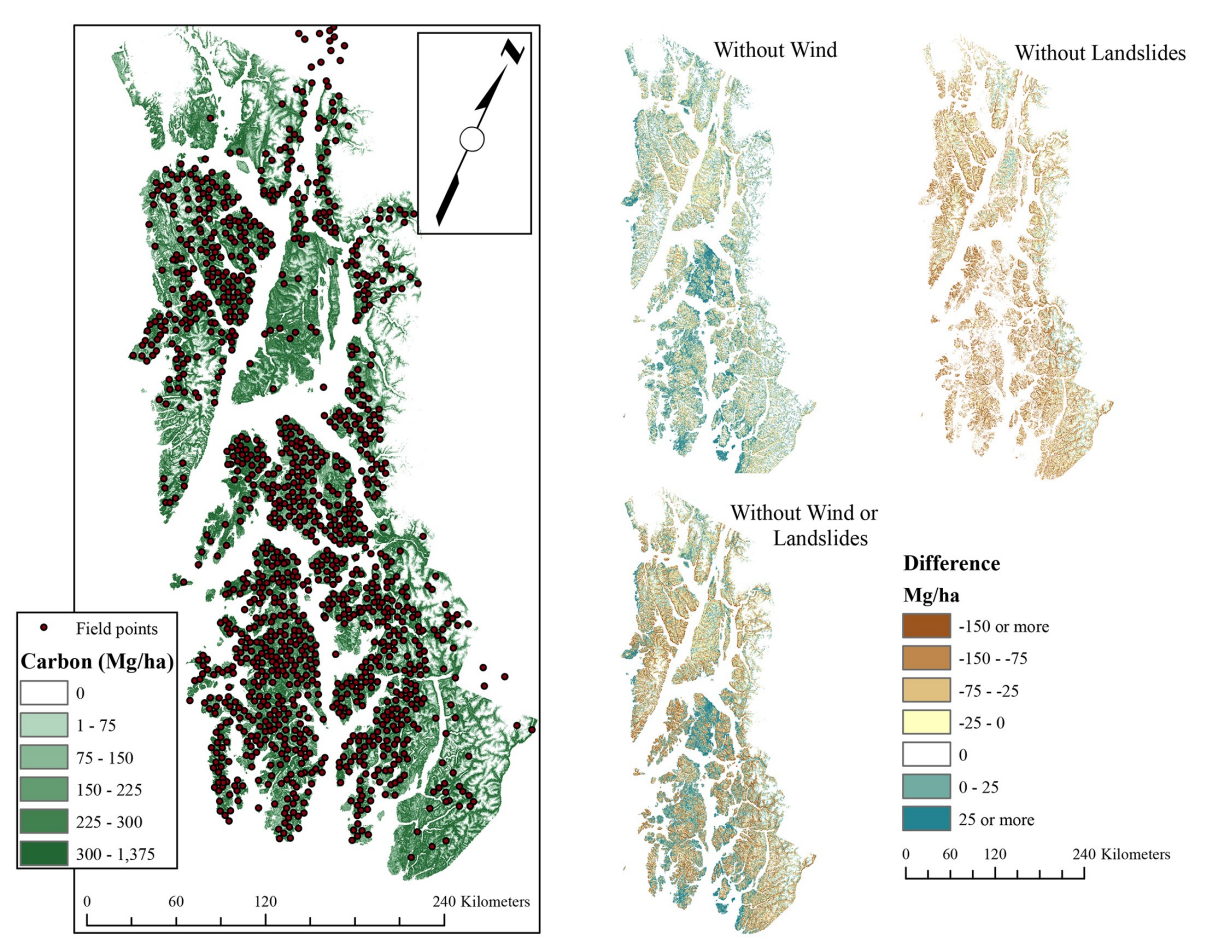
Modeled baseline C stocks and distribution of field points. Smaller regional maps show result of removing disturbance processes (via setting exposure to zero) and then differencing with modeled carbon stocks; negative values indicate more C when disturbances are included, positive values indicate less C.

Estimated potential biomass (living and dead) carbon ranged from 1.21–1.52 Pg (Table 1). Overall regional biomass did not change substantially (-2%) when the model was run assuming no wind disturbance. There were local differences, however, with many open, low angle areas predicted to have higher biomass (Fig 2). Removing the landslide component resulted in a substantial decline (-18 - -19%) in predicted biomass regionally, with a few areas of predicted increase in very steep topographical locations. Removing both disturbance processes resulted in a substantial decline (-15 - -16%) in regional carbon, but not as much as removing landslide alone. This suggests an interaction between wind and landslide exposure (Fig 2).

Basal area was higher in all non-disturbance scenarios, though to a lesser extent than C. In terms of individual stems, the removal of wind lowered estimated cumulative stem counts by a substantial margin, whereas removing landslides increased the number of stems. These two counteracting processes nearly canceled each other out when both disturbances were removed (Table 2). Together, these results suggest that wind exposure has little effect on carbon (potentially via the offsetting of lower biomass and higher tree density, though the multiple tests do not agree on strength); landslides have a stronger relationship. Though landslides appear to drive lower overall tree density (setting landslide exposure to zero results in higher cumulative stem values), their removal from the BA estimation reduces cumulative BA enough to also result in a significant decline in total biomass. Overall, both disturbances independently and combined result in higher biomass through changes to both BA and tree density.

**Table 2:** Estimated cumulative basal area and cumulative stems.

	Regional basal area (m^2^)	Difference from actual (%)	Cumulative stems (individuals)	Difference from actual (%)
Actual conditions	2.40 x 10^8^	-	7.49 x 10^9^	-
No wind	2.27 x 10^8^	-5	6.89 x 10^9^	-8
No slide	2.14 x 10^8^	-11	8.13 x 10^9^	+9
Neither	2.12 x 10^8^	-12	7.71 x 10^9^	+3

## Discussion

The goal of this work was to determine the significance of disturbances to regional C baselines in the most biomass-C dense forest biome on the planet, temperate rainforests. The region investigated, southeast Alaska, is known as a globally significant C storehouse [52, 54] though a high resolution, spatially explicit estimate of C stocks never been conducted. Spatial differences in disturbance exposure were correlated with baseline differences in biomass, tree density, and BA throughout the region, and those differences scaled up to the regional level. Generally, higher biomass was correlated with increasing disturbance exposure at the low end of the disturbance gradient, as hypothesized, even when accounting for the short-term variation imposed by random mortality. At the high end of the disturbance exposure gradient, very high wind exposures were not correlated with lower biomass C, but very high landslide exposures were associated with low (zero) estimates of biomass C. This is not surprising, as extremely steep slopes (e.g., >60^0^) are highly unstable and generally not vegetated as a result. In all cases, removing disturbance as a factor in the models resulted in lower overall biomass C, sometimes substantially. This was also true for BA. Tree density was mixed, with wind driving higher densities and landslides lower densities. This suggests that regionally, disturbances are maintaining higher levels of production in vegetated landscapes than would exist in their absence.

### Regional C stocks

Estimated potential regional biomass C stocks ranged from 1.21–1.52 Pg. This represents 5–7% of total US forest C (including public and private land, [41]). This is a potential C stock, not considering active or historical removal of C via logging. Logging has a history in the region; [52] estimated that between .01-.02 Pg were lost to logging between 1900–1995. Soil C, not included here, is a larger fraction of total ecosystem C than biomass. A regional modeling effort [55] estimated an average of 302 (+/- 146) Mg soil C ha^-1^, for a total of 1.78 Pg for the same region. Together, they add to a potential ecosystem C of 2.99–3.30 Pg C for the study area.

The estimate presented here align well with previous estimates in the general area, which ranged from 0.97–0.99 Pg of biomass C and 2.8 +/- 0.5 including soil C [52] and the FIA dataset, which estimated 0.863 Pg biomass C in forested areas only (>10% cover). The slightly lower value predicted by [52] is likely due to differences in design and slight differences in extent: This study focused on the potential natural C stocks for the region and thus excluded logging as a factor, whereas [52] incorporated logging. They also only estimated C stocks for the Tongass National Forest (68,000 km^2^), whereas this study did the entire region (76,492 km^2^). The FIA dataset is similarly estimating C over a smaller area (>10% only).

#### Wind

Across the landscape, the role of wind exposure was relatively minor compared to landslides, and the statistical results were mixed. In terms of a direct correlation, trees were denser in areas with higher wind exposure but that relationship did not extend to BA or biomass. The correlation with density, while significant, was very weak. No decline in biomass at high levels of wind exposure was found (using all 1491 plots to allow for the possibility of sparse/non-forest conditions due to wind; S1 Fig), suggesting that even in areas of highest exposure, the current wind regime does not drive significantly lower levels of baseline C at the regional scale. This implies that while wind disturbance may quite significant in terms of impact after discrete blowdown events [35], its influence at the regional scale is less important than other factors. In [35], at a finer scale (single island), exposure was calculated slightly differently: Wind exposure was incorporated into a broad, single index which included wind, soil stability, slope, and elevation. In the present study, those were treated as separate variables from wind exposure to allow for interactions between topography and the wind itself. The lack of a meaningful relationship between wind and biomass/BA (in terms of regional scale totals) suggests that the role of wind exposure in driving baseline differences in forest structure is more significant at finer, subregional scales and less important at broader scales (Fig 2). Generally, wind effect was strongest on lower slopes, where excluding wind resulted in slightly higher projected C; this was offset by the lower magnitude but widespread reduction in C on steeper slopes. The total effect is little net change in C upon removing wind exposure.

#### Landslides

In contrast to wind, landslide suitability was correlated more strongly with all structural variables and appeared to be more influential at the regional scale. In addition to potentially alleviating nutrient limitation (by exposing bedrock to weathering and shifting live biomass to the decomposing biomass pool), landslides also influence drainage and likely disrupt iron pan formation and Sphagnum introgression. Soil moisture is negatively correlated with forest structure (community composition and structure: [56]; biomass: [37]), as well as nutrient dynamics [57]. Most studies of the influence of landslide on soil properties have focused on fertility, with a decline in nutrient availability associated with the burial of surficial organic layers, e.g. [58], noted in some studies, or no difference noted between landslide deposits and undisturbed soils [59] immediately after a disturbance. However, others [60] noted that disturbances in areas where N-fixers are common post-disturbance community, as here, disturbances can progressively increase soil N availability. Over short timescales (<30 years), slower height growth on landslide scars relative to neighboring slopes was noted [61] in coastal forests to the south, however they did not distinguish between planted and naturally regenerating seedlings. They also noted that N-fixing species were a main component of the recovering ecosystem, potentially increasing long-term biomass, which is the focus here. In perhumid temperate rainforest systems, where drainage is highly correlated with productivity, changes in nutrient availability and drainage (associated with decreases in soil density in deposit zones) seem likely to encourage more productive forests in landslide susceptible locations [62]. On the other end of the disturbance exposure spectrum, a decline in biomass at high levels of landslide exposure is apparent (using all 1491 plots to allow for the possibility of sparse/non-forest conditions due to wind; S1 Fig). This is trivially true but nonetheless noted, as cliffs and very steep slopes are consistently moving, precluding forest establishment in many cases.

It is also possible that the drivers of landslide likelihood (steeper slopes) are partially confounding the relationship. Slope is strong driver of biomass at local scales [37], and significantly positively correlated with biomass at this broad scale, though weakly (forested plots only; p < 0.05, r^2^ = 0.06; square root transformed). That landslide susceptibility was retained in each independent test of the relationship (correlations, stepwise variable selection, and random forests) suggests that landslides themselves have different properties from purely slope-induced differences in drainage rates. At sub-regional scales, the effect of removing landslides was muted, with no change in areas where no landslides are possible (flat areas) and declines in C in steeper terrain (Fig 2). A second difficulty in landslide exposure is primarily related to landslide initiation rather than potential deposition. Landslides in SE Alaska are generally confined to steep slopes, even in their depositional area [38], however more intensive modeling of landslide movement downhill and across slopes is necessary to get a better estimate of exposed area.

#### Overall

The cumulative impact of both disturbances is a slight decrease in tree density and an increase in carbon and BA, based on modeled outcomes where disturbances were removed. The effect of the individual disturbance types partially offset each other at the regional scale, with wind correlated with higher tree densities in some locations countered by lower density, larger trees in other, more landslide exposed areas (Fig 2). In other words, the overall correlation of higher biomass with disturbance exposure is spatially heterogeneous when seen at finer scales. In the central portion of the region, where the topography is lower slope, disturbances (particularly wind) appear to be constraining biomass C, in that removing them from the model results in projected higher C values. In steeper locations, however, removing disturbances generally results in lower C, as those are the areas where landslides appear more significant. It should be noted that landslides are not independent of wind; there are higher landslide probabilities in wind exposed locations. This has been attributed to shaking of the trees during high rain/storm events [39, 48]. This interaction adds further nuance to the disturbance discussion and suggests that treating the individual disturbances separately also requires considering their interactions as a separate mechanistic component of the overall regime. In sum, even within an ecosystem type individual disturbance processes vary in their spatial impacts and therefore in their correlation with baseline C.

This correlation between higher disturbance rates and higher biomass is strikingly different from further south in the seasonal temperate rainforest (where fire is a factor and can impact broad-scale C for centuries; [63]) where disturbance rates maintain lower baseline C than would be expected without disturbance [64]. In that case, suppression of disturbances would be expected to raise C stocks, though unlikely to be sustainable. In this perhumid portion of the temperate rainforest, however, disturbance frequency and extents are some of the lowest of all forested ecoregions in North America (<0.5% per year, [27]). It appears that while there is some finer scale heterogeneity in the effects of increasing disturbance exposure (Fig 2), the net effect is higher biomass in areas of higher exposure, and as a result, higher predicted C stocks.

### Limitations

Like most broad-scale regional studies, the results here are correlative rather than necessarily causal. A counter hypothesis would be that disturbance exposure is higher in areas that are also prone to higher biomass forests for other reasons. The mechanisms that would drive higher biomass in these exposed areas independent of disturbance would be likely be aspect (via solar exposure) and slope (via better drainage) for wind and landslide disturbances respectively. Both aspect and slope were considered explicitly alongside the disturbance variables, and the disturbance exposure variables were retained in the statistical models. Second, aspect and slope are not perfectly correlated with wind and landslides; wind exposure is modified by upwind topography, so there are sheltered southeast facing slopes (for example). Similarly, there are steep slopes with relatively low landslide probability because local topography does not concentrate drainage. Further fine scale work would be valuable, especially dendrochronological or paleoecological studies which could link specific disturbance histories at a point to stand biomass metrics. To our knowledge, there are no long-term (1000+ year) studies on landslide succession, which would be valuable comparisons to existing long-term chronosequences (generally post-glacial, [16]). This is often challenging in this environment due to heart rot making precise dating impossible and the difficulty of ascribing the cause of stand initiation several thousand years later. Nonetheless, the consistent association of higher baseline biomass and higher exposures regardless of recent disturbance history, coupled with undisturbed chronosequences from the region (Glacier Bay) which show substantial biomass declines in undisturbed areas [14], suggests that productivity is enhanced by infrequent but not non-existent disturbance regimes.

## Conclusions

The temperate rainforests of southeast Alaska are some of the most biomass-C rich forests in the world and can potentially contain C stores equivalent to 5–7% of the lower 48 forest C stocks in biomass pools alone. Higher C, contained in lower density but larger tree stands, was associated with higher exposure to infrequent disturbance (wind and landslide) processes; areas sheltered from those disturbances had lower values (lower BA, lower biomass). Landslides were more associated with higher biomass C than wind exposure, which was more associated with higher tree densities. The strength of the cumulative action of these two disturbance processes was heterogeneous in space, and at finer scales some areas had lower predicted biomass C. This is consistent with the hypothesis that in very infrequently disturbed systems, occasional mortality events result in more productive stands at the landscape and regional scale. While any disturbed location will lose C as a result of that event in the short term, the overall, broad-scale relationship in this high C density, infrequently disturbed system is higher baseline C in areas of higher wind and landslide exposure.

## Supporting information

S1 Fig Direct correlations between disturbance and biomass. Wind exposure and landslide exposure compared to the biomass variables: Tree density, biomass, and basal area. Red line represents a simple linear regression to show trends.
(PDF)

## References

[1] Pickett STA, Kolasa J, Armesto JJ, Collins SL. The ecological concept of disturbance and its expression at various hierarchical levels. Oikos. 1989; 54(2): 129–136.

[2] White PS, Jentsch A. The search for generality in studies of disturbance and ecosystem dynamics In Progress in Botany 2001: 399–450. Springer, Berlin, Heidelberg.

[3] Running SW. Ecosystem disturbance, carbon, and climate. Science. 2008; 321(5889): 652–653. 10.1126/science.1159607 18669853

[4] Amiro BD, Barr AG, Barr JG, Black TA, Bracho R, Brown M, et al Ecosystem carbon dioxide fluxes after disturbance in forests of North America. Journal of Geophysical Research: Biogeosciences. 2010; 115(G4).

[5] Thom D, Seidl R. Natural disturbance impacts on ecosystem services and biodiversity in temperate and boreal forests. Biological Reviews. 2016; 91(3): 760–781. 10.1111/brv.12193 26010526 PMC4898621

[6] Dantas VDL, Hirota M, Oliveira RS, Pausas JG. Disturbance maintains alternative biome states. Ecology Letters. 2016; 19(1): 12–19. 10.1111/ele.12537 26493189

[7] Rhemtulla JM, Mladenoff DJ, Clayton MK. Historical forest baselines reveal potential for continued carbon sequestration. Proceedings of the National Academy of Sciences. 2009; 106(15): 6082–6087. 10.1073/pnas.0810076106 PMC2669390 19369213

[8] Buma B. Disturbance interactions: Characterization, prediction, and the potential for cascading effects. Ecosphere. 2015; 6:art70. 10.1890/ES14-00365.1

[9] Morris JL, DeRose RJ, Brunelle AR. Long-term landscape changes in a subalpine spruce-fir forest in central Utah, USA. Forest Ecosystems. 2015; 2(1): 35.

[10] Paine RT, Tegner MJ, Johnson EA. Compounded perturbations yield ecological surprises. Ecosystems. 1998; 1(6): 535–545.

[11] McLauchlan KK, Higuera PE, Gavin DG, Perakis SS, Mack MM, Alexander H, et al Reconstructing disturbances and their biogeochemical consequences over multiple timescales. BioScience. 2014; 64(2): 105–116.

[12] Kranabetter JM, McLauchlan KK, Enders SK, Fraterrigo JM, Higuera PE, Morris JL, et al A framework to assess biogeochemical response to ecosystem disturbance using nutrient partitioning ratios. Ecosystems. 2016; 19: 387–395.

[13] Hudiburg TW, Higuera PE, Hicke JE. Fire-regime variability impacts forest carbon dynamics for centuries to millennia. Biogeosciences. 2017; 14: 3873–3882.

[14] Wardle DA, Walker LR, Bardgett RD. Ecosystem properties and forest decline in contrasting long-term chronosequences. Science. 2004; 305(5683): 509–513. 10.1126/science.1098778 15205475

[15] Parfitt RL, Ross DJ, Coomes DA, Richardson SJ, Smale MC, Dahlgren RA. N and P in New Zealand soil chronosequences and relationships with foliar N and P. Biogeochemistry. 2005; 75(2): 305–328.

[16] Peltzer DA, Wardle DA, Allison VJ, Baisden WT, Bardgett RD, Chadwick OA, et al Understanding ecosystem retrogression. Ecological Monographs, 2010; 80(4): 509–529.

[17] Simard M, Lecomte N, Bergeron Y, Bernier PY, Paré D. Forest productivity decline caused by successional paludification of boreal soils. Ecological Applications. 2007; 17(6): 1619–1637. 17913128 10.1890/06-1795.1

[18] Matson PA, Boone RD. Natural Disturbance and Nitrogen Mineralization: Wave‐Form Dieback of Mountain Hemlock in the Oregon Cascades. Ecology. 1984; 65(5): 1511–1516.

[19] Binkley D, Smith FW, Son Y. Nutrient supply and declines in leaf area and production in lodgepole pine. Canadian Journal of Forest Research. 1995; 25: 621–628.

[20] Hobbie SE, Nadelhoffer KJ, Högberg P. A synthesis: the role of nutrients as constraints on carbon balances in boreal and arctic regions. Plant and Soil. 2002; 242(1): 163–170.

[21] Ugolini FC, Mann DH. Biopedological origin of peatlands in southeast Alaska. Nature. 1979; 281: 366–368

[22] Klinger LF, Elias SA, Behan‐Pelletier VM, Williams NE. The bog climax hypothesis: fossil arthropod and stratigraphic evidence in peat sections from southeast Alaska, USA. Ecography. 1990; 13(1): 72–80.

[23] Vitousek PM, Porder S, Houlton BZ, Chadwick OA. Terrestrial phosphorus limitation: mechanisms, implications, and nitrogen–phosphorus interactions. Ecological Applications. 2010; 20(1): 5–15 20349827 10.1890/08-0127.1

[24] Perakis SS, Tepley AJ, Compton JE. Disturbance and topography shape nitrogen availability and δ15N over long-term forest succession. Ecosystems. 2015; 18(4): 573–588.

[25] Hoffman KM, Trant AJ, Nijland W, Starzomski BM. Ecological legacies of fire detected using plot-level measurements and LiDAR in an old growth temperate rainforest. Forest Ecology and Management. 2018; 424: 11–20.

[26] Turner MG, Gardner RH, O'Neill RV. Landscape ecology in theory and practice: pattern and process. 2015 Springer New York, New York, USA.

[27] Buma B, Costanza JK, Riitters K. Determining the size of a complete disturbance landscape: Multi-scale, continental analysis of forest change. Environmental Monitoring and Assessment. 2017; 189: 642 10.1007/s10661-017-6364-x 29164343

[28] Baichtal JF, Crockford SJ, Carlson RK. Possible evidence of warmer, drier climates during the early Holocene of southern southeast Alaska from shell-bearing raised marine and peat deposits. 2008. AAA annual meeting, poster session.

[29] Hard JS. The forest ecosystem of southeast Alaska: 2. Forest insects *Gen*. *Tech*. *Rep*. *PNW-GTR-013*. Portland, OR: US Department of Agriculture, Forest Service, Pacific Northwest Research Station 1974.

[30] Hennon PE, D'Amore DV, Schaberg PG, Wittwer DT, Shanley CS. Shifting climate, altered niche, and a dynamic conservation strategy for yellow-cedar in the North Pacific coastal rainforest. BioScience. 2012; 62(2): 147–158.

[31] Buma B, Hennon PE, Harrington CA, Popkin JR, Krapek J, Lamb MS, et al Emerging climate‐driven disturbance processes: widespread mortality associated with snow‐to‐rain transitions across 10° of latitude and half the range of a climate‐threatened conifer. Global Change Biology. 2017; 23(7): 2903–2914. 10.1111/gcb.13555 27891717

[32] Buma B, Barrett TM. Spatial and topographic trends in forest expansion and biomass change, from regional to local scales. Global Change Biology. 2015; 21(9): 3445–3454. 10.1111/gcb.12915 25726931

[33] Boose ER, Chamberlin KE, Foster DR. Landscape and regional impacts of hurricanes in New England. Ecological Monographs. 2001; 71(1): 27–48.

[34] Harris AS. Wind in the forests of southeast Alaska and guides for reducing damage Gen. Tech. Rep. PNW-GTR-244. Portland, OR: US Department of Agriculture, Forest Service, Pacific Northwest Research Station 63 1999.

[35] Kramer MG, Hansen AJ, Taper ML, Kissinger EJ. Abiotic controls on long‐term windthrow disturbance and temperate rain forest dynamics in southeast Alaska. Ecology. 2001; 82(10): 2749–2768.

[36] Kramer MG, Sollins P, Sletten RS. Soil carbon dynamics across a windthrow disturbance sequence in southeast Alaska. Ecology. 2004; 85(8): 2230–2244.

[37] Buma B, Krapek J, Edwards RT. Watershed-scale forest biomass distribution in a perhumid temperate rainforest as driven by topographic, soil, and disturbance variables. Canadian Journal of Forest Research. 2016; 46(6): 844–854.

[38] Swanston DN. The forest ecosystem of southeast Alaska: 5. Soil mass movement *Gen*. *Tech*. *Rep*. *PNW-GTR-017*. Portland, OR: US Department of Agriculture, Forest Service, Pacific Northwest Research Station 17 1974.

[39] Diaz-Yanez O, Mola-Yudego B, Gonzalez-Olabarria JR, Pukkala T. How does forest composition and structure affect the stability against wind and snow? Forest Ecology and Management. 2017; 401: 215–222.

[40] Kobayashi Y, Mori AS. The potential role of tree diversity in reducing shallow landslide risk. Environmental Management. 2017; 59(25): 807–815. 28110357 10.1007/s00267-017-0820-9

[41] USFS. The Forest Inventory and Analysis Database: Database Description and Users Guide for Phase 2. Version 7.0.1. National Forest Inventory and Analysis Program, USDA Forest Service. 2018.

[42] Houghton JT, Meira Filho LG, Lim B, Treanton K, Mamaty I, et al; Callander BA, eds. 1997 Revised 1996 Intergovernmental Panel on Climate Guidelines for National Greenhouse Inventories. Paris: IPCC/OECD/IEA.

[43] Lutz JA, Larson AJ, Freund JA, Swanson ME, Bible KJ. The importance of large-diameter trees to forest structural heterogeneity. PLoS One. 2013; 8(12): e82784 10.1371/journal.pone.0082784 24376579 PMC3869720

[44] Moisen GG, Frescino TS. Comparing five modelling techniques for predicting forest characteristics. Ecological Modelling. 2002; 157(2–3): 209–225.

[45] Scenarios Network for Arctic Planning. University of Alaska. www.snap.uaf.edu. 2017. Retrieved 1 Sept 2017.

[46] Sexton JO, Song X-P, Feng M, Noojipady P, Anand A, Huang C, et al Global, 30-m resolution continuous fields of tree cover: Landsat-based rescaling of MODIS Vegetation Continuous Fields with lidar-based estimates of error. International Journal of Digital Earth. 2013; 130321031236007. 10.1080/17538947.2013.786146

[47] Ager AA, Vaillant NM, Finney MA, Preisler HK. Analyzing wildfire exposure and source–sink relationships on a fire prone forest landscape. Forest Ecology and Management. 2012; 267: 271–283.

[48] Buma B, Johnson AC. Disturbance interactions mediated by topography: Wind exposure, landslide susceptibility, and yellow cedar decline in southeast Alaskan temperate rainforests. Geomorphology. 2015; 228, 504–511.

[49] Rejou-Mechain M, Muller-Landau HC, Detto M, Thomas SC, Toan TL, Saatchi SS, et al Local spatial structure of forest biomass and its consequences for remote sensing of carbon stocks. Biogeosciences Discussions. 2014; 11: p.5711.

[50] Breiman L. Random forests. Machine Learning. 2001; 45(1): 5–32.

[51] Zhang G, Lu Y. Bias-corrected random forests in regression. Journal of Applied Statistics. 2012; 39(1): 151–160.

[52] Leighty WW, Hamburg SP, Caouette J. Effects of management on carbon sequestration in forest biomass in southeast Alaska. Ecosystems. 2006; 9(7), 1051–1065.

[53] Bond WJ, Woodward FI, Midgley GF. The global distribution of ecosystems in a world without fire. New Phytologist. 2005; 165(2): 525–538. 10.1111/j.1469-8137.2004.01252.x 15720663

[54] Keith H, Mackey BG, Lindenmayer DB. Re-evaluation of forest biomass carbon stocks and lessons from the world's most carbon-dense forests. Proceedings of the National Academy of Sciences. 2009; 106(28): 11635–11640. 10.1073/pnas.0901970106 PMC2701447 19553199

[55] McNicol G, Bulmer C, D’Amore DV, Sanborn P, Saunders S, Giesbrecht I, et al Large, climate-sensitive soil carbon stocks in the North Pacific coastal temperate rainforest. Environmental Research Letters. 2019; 14: 1.

[56] Bisbing SM, Cooper DJ, D'Amore DV, Marshall KN. Determinants of conifer distributions across peatland to forest gradients in the coastal temperate rainforest of southeast Alaska. Ecohydrology. 2016; 9(2): 354–367.

[57] Bisbing SM, D’Amore DV. Nitrogen dynamics vary across hydrologic gradients and by forest community composition in the perhumid coastal temperate rainforest of southeast Alaska. Canadian Journal of Forest Research. 2017; 48(2): 180–191.

[58] Wilcke W, Valladarez H, Stoyan R, Yasin S, Valarezo C, Zech W. Soil properties on a chronosequence of landslides in montane rain forest, Ecuador. Catena. 2003; 53(1): 79–95.

[59] Adams PW, Sidle RC. Soil conditions in three recent landslides in southeast Alaska. Forest Ecology and Management. 1987; 18(2): 93–102.

[60] Perakis SS, Sinkhorn ER, Compton JE. δ 15 N constraints on long-term nitrogen balances in temperate forests. Oecologia. 2011; 167(3): 793–807. 10.1007/s00442-011-2016-y 21614618

[61] Miles DWR, Swanson FJ, Youngberg CT. Effects of Landslide Erosion on Subsequent Douglas-fir Growth and Stocking Levels in the Western Cascades, Oregon. Soil Science Society of America Journal. 1984; 48(3), 667–671.

[62] Geertsema M, Pojar JJ. Influence of landslides on biophysical diversity—a perspective from British Columbia. Geomorphology. 2007; 89(1–2): 55–69.

[63] Hudiburg T, Law B, Turner DP, Campbell J, Donato D, Duane M. Carbon dynamics of Oregon and Northern California forests and potential land‐based carbon storage. Ecological Applications. 2009; 19(1): 163–180. 19323181 10.1890/07-2006.1

[64] Smithwick EA, Harmon ME, Remillard SM, Acker SA, Franklin JF. Potential upper bounds of carbon stores in forests of the Pacific Northwest. Ecological Applications. 2002; 12(5): 1303–1317.

