# Supplementary material for: Long-term exposure to more frequent disturbances increases baseline carbon in some ecosystems: Mapping and quantifying the disturbance frequency-ecosystem C relationship
Source: PLoS One. 2019 Feb 21;14(2):e0212526. doi: 10.1371/journal.pone.0212526 (PMC6383921; doi:10.1371/journal.pone.0212526)
Supplement: S1 Fig — Wind exposure and landslide exposure compared to the biomass variables: Tree density, biomass, and basal area. Red line represents a simple linear regression to show trends. (PDF) [file pone.0212526.s001.pdf]

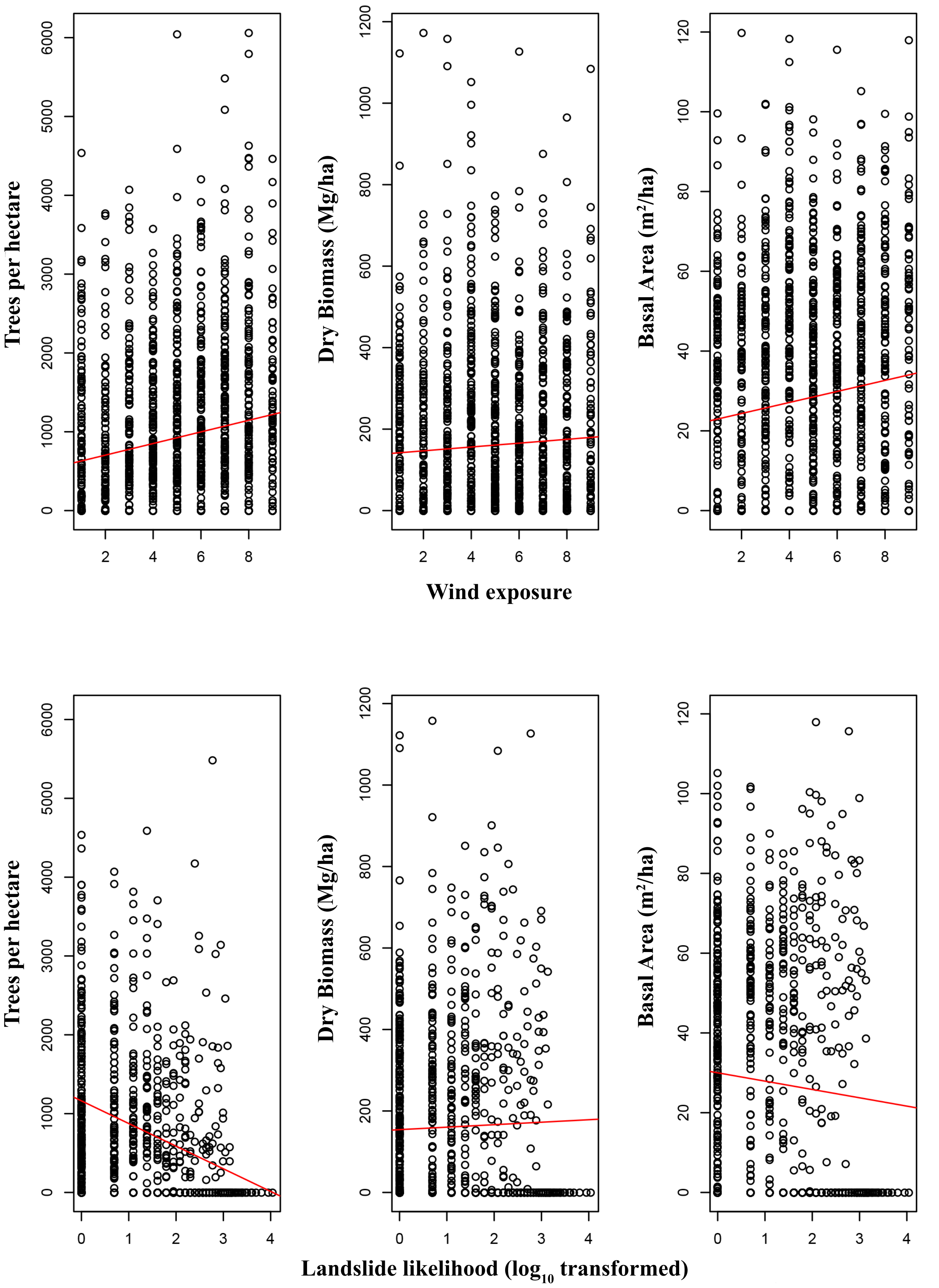

Trees per hectare
Dry Biomass (Mg/ha)
Basal Area (m²/ha)
Wind exposure
Trees per hectare
Dry Biomass (Mg/ha)
Basal Area (m²/ha)
Landslide likelihood (log10 transformed)
